# Photothermal Effect and Biomineralization of Black Phosphorus Nanosheet‐Composited Hydrogel Boosts Synergistic Treatment of Dentin Hypersensitivity

**DOI:** 10.1002/advs.202412561

**Published:** 2025-01-03

**Authors:** Qihui Wang, Guoliang Wang, Xinru Li, Di Li, Congxiao Zhang, Jianxun Ding

**Affiliations:** ^1^ Department of Stomatology The First Hospital of Jilin University 1 Xinmin Street Changchun 130061 P. R. China; ^2^ Key Laboratory of Polymer Ecomaterials Changchun Institute of Applied Chemistry Chinese Academy of Sciences 5625 Renmin Street Changchun 130022 P. R. China; ^3^ Department of Hepatobiliary and Pancreatic Surgery General Surgery Center The First Hospital of Jilin University 1 Xinmin Street Changchun 130061 P. R. China; ^4^ School of Applied Chemistry and Engineering University of Science and Technology of China 96 Jinzhai Road Hefei 230026 P. R. China

**Keywords:** alleviating toothache, biomineralization, black phosphorus nanosheet, dentin hypersensitivity, gelatin methacryloyl hydrogel, photothermal effect

## Abstract

Dentin hypersensitivity (DH), marked by exposed dentinal tubules, presents as a sharp toothache triggered by stimuli and subsides when the stimuli are removed. To address the limitations of current commercial desensitizers in terms of acid resistance, friction resistance, and stability, a black phosphorus nanosheet‐composited methacrylate gelatin hydrogel (GelMA/BP) is developed for DH treatment, leveraging the synergistic effects of photothermal therapy and biomineralization. Incorporating the BP nanosheet provided GelMA/BP with a stable photothermal response and the continuous release of phosphate anions, which blocked dentinal tubules by converting light energy into heat and initiating biomineralization. In vitro desensitizing therapy showed that the dentinal tubule diameter in the GelMA/BP50 group (0−1.13 µm) is significantly reduced compared to that in the DH‐model group (0−3.14 µm). The GelMA/BP50 group achieved an 86% tubule occlusion rate, with acid resistance of 80%, friction resistance of 76%, and long‐term stability of 74%. In vivo studies further validated the efficacy of GelMA/BP50, showing a reduction in tubule diameter (0−0.37 µm) and an occlusion rate of 79%, which alleviated toothache and increased intake and weight. These results demonstrate that this desensitizing hydrogel acts as an effective dentinal tubule sealant, offering promising clinical benefits for the topical treatment of DH.

## Graphical abstract

An advanced black phosphorus nanosheet‐composited methacrylate gelatin hydrogel is developed for the treatment of dentin hypersensitivity through the synergistic photothermal effect and biomineralization, which occluded 79% of dentinal tubules in vivo, with the remaining unsealed tubules narrowing to a diameter of 0−0.37 µm and alleviating toothache, demonstrating a promising desensitizing effect.

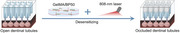

## 1 Introduction

Dentin hypersensitivity (DH) is a prevalent dental disease resulting from enamel integrity destruction and dentin exposure, which presents as a transient toothache triggered by changes in temperature (cold or hot), exposure to chemical substances (sour or sweet), and mechanical stimuli (friction or mastication).^[^ ^1^, ^2^ ^]^ Epidemiological studies indicate that 41.9% of Europeans aged 18 to 35 suffered toothache in response to dental stimuli, compared to 25.5% of Chinese individuals aged 20 to 69.^[^ ^3^, ^4^ ^]^ Numerous desensitizing approaches, such as ion or protein precipitation, bionic remineralization, nerve blockade, and photo‐biomodulation, are proposed to occlude exposed dentinal tubules and alleviate DH.^[^ ^5^ ^]^ However, current desensitizing strategies generally suffer from limitations, such as inadequate acid resistance, limited abrasion resistance, and poor stability, potentially resulting in dentin cracks and even dental pulp injury.^[^ ^6^ ^]^ In clinical therapy, limited duration of effectiveness, inconsistent results among patients, adverse reactions to certain ingredients in the desensitizing agents, complex application procedures, and interference with the bonding process of dental restorations limit the application of desensitizers.

To address these challenges, researchers have investigated the use of lasers to block dentinal tubules, primarily by elevating temperature of the dentin surface. This heating process leads to melting of the dentin, which reduces diameters of the dentinal tubules.^[^ ^7^ ^]^ Although laser‐based occlusion shows promise, its efficacy as a standalone treatment remains suboptimal.^[^ ^6^ ^]^ Thus, there is a pressing need to develop synergistic laser desensitization techniques to enhance the effectiveness of DH treatment. Photothermal therapy (PTT), characterized by minimal invasiveness, no drug resistance, and low systemic toxicity, produces photothermal effects exclusively in the simultaneous presence of both lasers and photothermal agents (PTAs).^[^ ^8^ ^]^ In recent years, PTT has emerged as an innovative therapeutic strategy, successfully eliminating *Streptococcus mutans* colonies in the oral cavity,^[^ ^9^ ^]^ demonstrating significant potential in managing dental caries owing to various etiologies.^[^ ^10^ ^]^ Laser desensitization technology achieves a reduction in the diameters of dentinal tubules by raising surface temperature of the dentin to melt it. To prevent dentin cracks caused by thermal damage and insufficient ability to store thermal energy, the black phosphorus (BP) nanosheet‐composited methacrylate gelatin (GelMA/BP) hydrogel was designed for dental PTT application.

**Scheme 1:**
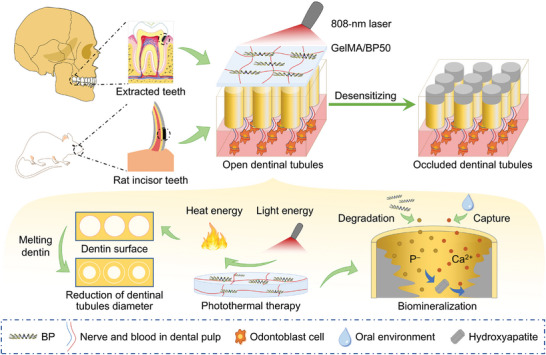
Construction process and desensitizing treatment mechanisms of DH.

In this study, the effectiveness of advanced GelMA/BP hydrogel was systematically evaluated for dental desensitization. During desensitization, GelMA/BP50 converted light energy into heat, effectively sealing the dentinal tubules by melting superficial dentin. Simultaneously, GelMA/BP50 continuously released phosphate anions, that is, H_2_PO_4_^−^, HPO_4_^2−^, and PO_4_^3−^ (unified labeling as P^−^) and captured calcium ion (Ca^2+^), facilitating the formation of hydroxyapatite (HA)‐like analog within the dentinal tubules and promoting dentin biomineralization (**Scheme** **1**).^[^ ^11^ ^]^ Researchers have demonstrated that the in‐depth remineralization therapy within deep dentinal tubules for DH is highly effective and produces significant long‐term effects, demonstrating considerable potential for applications in hard tissue repair.^[^ ^12^ ^]^ In this experiment, GelMA/BP sealed dentinal tubules and demonstrated strong resistance to acid, friction, and external stimuli. As a result, toothache triggered by minor dental pulp stimuli was alleviated after desensitizing treatment. These findings provide valuable insights into the development of more effective clinical treatments for DH.

## 2 Results and Discussion

### 2.1 Synthesis and Characterizations of Black Phosphorus Nanosheet

**Figure 1:**
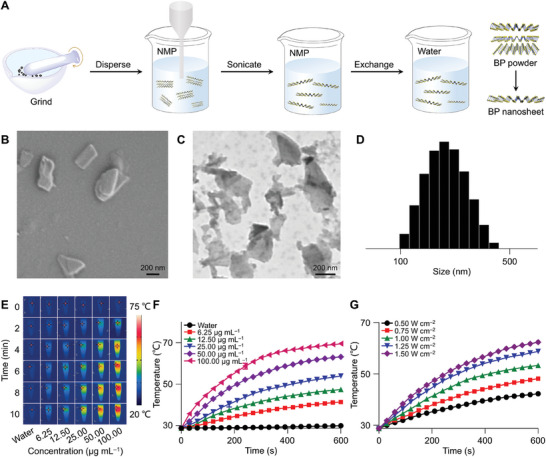
Preparation and characterizations of BP nanosheet. A) BP nanosheet prepared by liquid exfoliation. B,C) FE‐SEM (B) and TEM images (C) of BP nanosheet. D) *D*_h_ of BP nanosheet. E) Infrared images of BP suspension under laser irradiation. F) Temperature curves of different BP nanosheet concentrations under 1.0 W cm^−2^ laser irradiation. G) Temperature curves of 25.0 µg mL^−1^ BP nanosheet suspension under different laser irradiation power.

BP, a 2D inorganic material, attracted widespread attention in PTT owing to its excellent biocompatibility, exceptional photothermal stability, and high photothermal conversion efficiency.^[^ ^13^ ^]^ As shown in **Figure** **1**A, BP nanosheet was prepared by liquid exfoliation and characterized using field emission scanning electron microscope (FE‐SEM) and transmission electron microscopy (TEM) (Figure 1B,C). Previous research findings reported that the diameters of dentinal tubules typically range from 0.9 to 2.5 µm.^[^ ^14^ ^]^ The average length of BP nanosheet was 203.0 ± 61.3 nm (Figure 1D), characterized by dynamic light scattering (DLS), which was significantly smaller than the diameters of dentinal tubules, allowing deeper penetration and enhanced desensitization.

Near‐infrared (NIR) light at specific wavelengths induces PTAs to transition from their ground state to an excited state, absorbing light energy and converting it into heat, then returning to the ground state, thus facilitating PTT. Effective PTAs require high photothermal conversion efficiency for successful PTT. Consequently, the photothermal conversion capabilities of BP nanosheet in various concentrations were assessed (Figure 1E−G). At a constant laser power of 1 W cm^−2^, the temperature increases of BP nanosheet suspension correlated positively with concentration. After 10 min of laser irradiation, the temperature of pure water rose by only 1.2 °C, while the temperature of the 25.0 µg mL^−1^ BP nanosheet suspension increased by 25.2 °C, indicating its excellent photothermal effect. Moreover, at a fixed BP nanosheet concentration of 25.0 µg mL^−1^, the temperature rise positively correlated with laser power. To assess photothermal stability, a 25.0 µg mL^−1^ BP nanosheet suspension was exposed to a laser power of 1.0 W cm^−2^. As shown in Figure S1A (Supporting Information), the BP nanosheet suspension maintained a consistent temperature fluctuation pattern during four cycles of on/off laser irradiation, indicating that the BP nanosheet was thermally stable across multiple photothermal conversion cycles.

Efficient and safe PTT effectively enhances the integration of hard tissues without causing damage to surrounding tissues.^[^ ^15^ ^]^ Photothermal conversion efficiency plays a crucial role in the therapeutic efficacy of PTT. Numerous PTAs with different photothermal conversion rates have been applied in PTT, with reported efficiencies of 48.5% for Au@MOF, 27.7% for glycol‐ and polyethyleneimine‐modified gold nanorods (mPEG‐PEI‐AuNRs), and 34.5% for diketopyrrolopyrrole−triphenylamine (DPP−PTA).^[^ ^16^ ^]^ Our investigation showed that BP nanosheet achieved a notably high photothermal conversion efficiency of 79.4% (*η* = 79.4%), as determined by the temperature rise‐decline curve (Figure S1B, Supporting Information) and photothermal fitting curve (Figure S1C, Supporting Information). This exceptional efficiency is expected to significantly enhance PTT performance, leading to faster sealing of dentin tubules and shorter treatment times.

### 2.2 Preparation and Characterizations of Black Phosphorus Nanosheet‐Composited Methacrylate Gelatin Hydrogel

**Figure 2:**
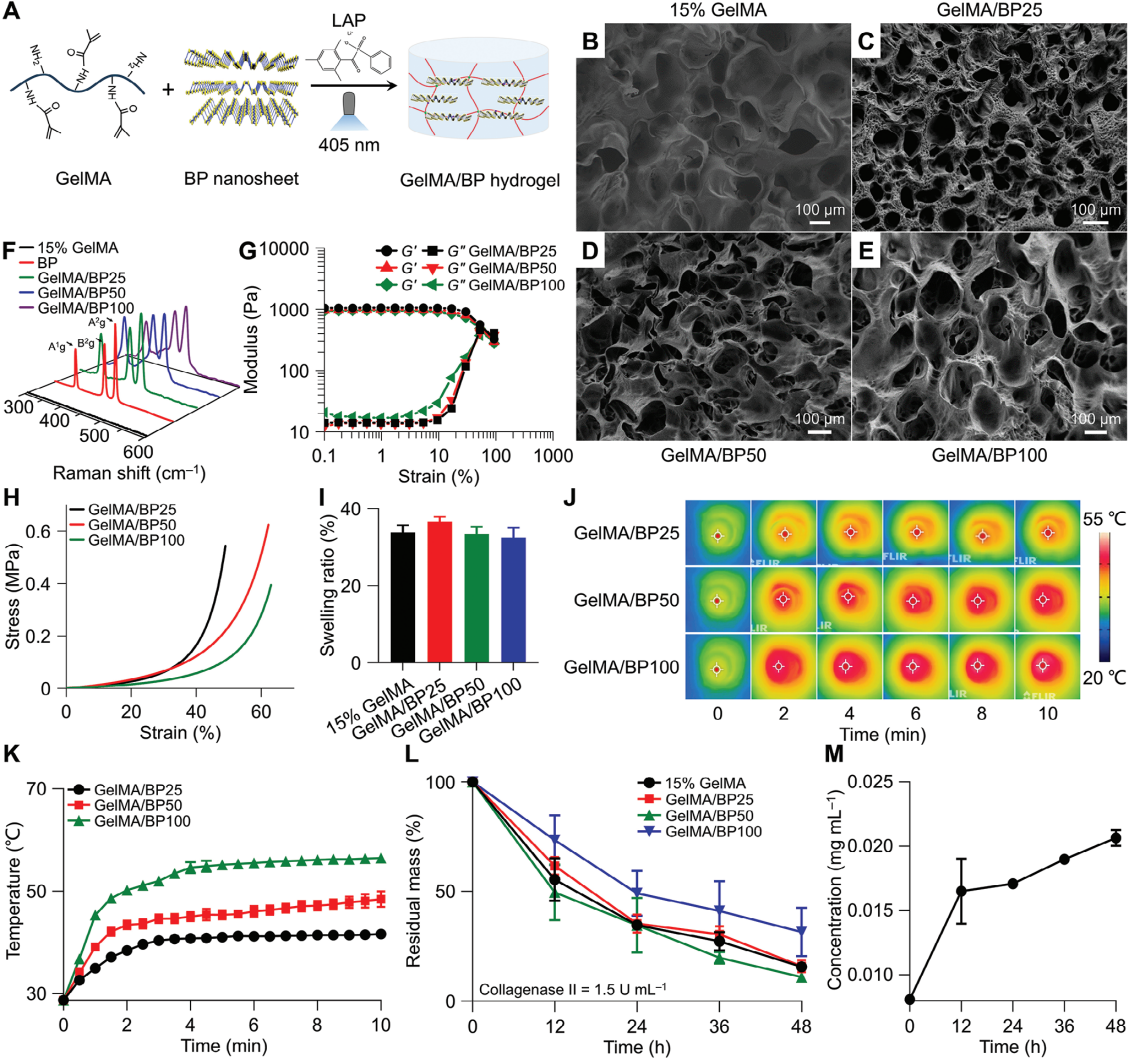
Preparation and characterizations of GelMA/BP. A) GelMA/BP construction. B−E) FE‐SEM images of 15% (*W*/*V*) GelMA hydrogel (B), GelMA/BP25 hydrogel (C), GelMA/BP50 hydrogel (D), and GelMA/BP100 hydrogel (E). F) Raman spectra of BP nanosheet and GelMA/BP. G) Amplitude scanning strain results of GelMA/BP. H) Strain−stress curves of GelMA/BP. I) Swelling ratio of GelMA/BP. J,K) Infrared images (J) and temperature curves (K) of GelMA/BP. L,M) A percentage of residual mass (L) and P release capacity (M) of GelMA/BP.

GelMA has demonstrated potential in promoting dental pulp stem cell regeneration, calcium deposition, and osteogenic differentiation.^[^ ^17^, ^18^ ^]^ The GelMA hydrogel matrix, fortified with Panax notoginseng saponin R1, was employed to effectively facilitate the odontogenic differentiation of mouse dental papilla cells, demonstrating a potent capacity to enhance the generation of restorative dentin.^[^ ^19^ ^]^ Meanwhile, GelMA hydrogel efficiently loaded cells and adhered to the dentin tubule wall, promoting the secretion of a matrix resembling reparative dentin.^[^ ^20^ ^]^ Combining GelMA's excellent capabilities with laser‐induced desensitization, a GelMA/BP was designed for DH therapy, characterized by its straightforward preparation process, superior photothermal attributes, and enhanced desensitizing effect compared to other hydrogels.^[^ ^21^, ^22^ ^]^ As shown in **Figure** **2**A, GelMA/BP was synthesized via photo‐cross‐linking (405 nm blue light) using lithium phenyl‐2,4,6‐trimethylbenzoylphosphinate (LAP) as the photoinitiator. BP nanosheet suspensions at concentrations of 25.0, 50.0, and 100.0 µg mL^−1^, known for their significant photothermal effect under laser irradiation, were incorporated into the hydrogel formulation. Mechanical testing of 10% and 15% (*W*/*V*) GelMA hydrogels revealed that 15% (*W*/*V*) GelMA exhibited optimal strength and deformability (Figure S2A,B, Supporting Information). Therefore, GelMA/BP was prepared using 15% (*W*/*V*) GelMA and three BP nanosheet concentrations labeled GelMA/BP25, GelMA/BP50, and GelMA/BP100, respectively. The surface morphologies of GelMA and GelMA/BP hydrogels were observed via FE‐SEM (Figure 2B−E). GelMA exhibited a porous structure with an average pore size of 82.81 nm, while GelMA/BP100, GelMA/BP50, and GelMA/BP25 displayed a more compact porous structure with an average pore size of 75.29, 51.85, and 50.61 nm, respectively. The porous architecture facilitates the diffusion of ions, nutrients, and gases into the deeper layers of dentinal tubules, which influences a desensitizing effect.^[^ ^23^ ^]^ The addition of BP nanosheet increased the compactness of the porous structure, reduced pore size, and potentially contributed to the morphological changes during lyophilization.^[^ ^7^ ^]^

Raman spectroscopy confirmed the successful incorporation of BP nanosheet into the hydrogel. GelMA/BP exhibited characteristic BP nanosheet peaks, corresponding to the out‐of‐plane phonon mode A^1^g at 360.10 cm^−1^ and the in‐plane phonon modes B^2^g and A^2^g at 437.89 and 464.23 cm^−1^, respectively, while GelMA alone lacked these characteristic peaks (Figure 2F). Mechanical properties of GelMA/BP were analyzed, revealing comparable amplitude scanning strain behavior across the different BP nanosheet concentrations (Figure 2G). The storage modulus (*G*′) and loss modulus (*G*″) showed no significant variation with increased BP nanosheet concentration, although the strain capacities increased, indicating favorable mechanical deformation properties (Figure 2H). Interestingly, GelMA/BP50 exhibited better stress strength properties than GelMA/BP100, possibly due to limited binding sites, reduced cross‐linked density, and stress points originating from the BP nanosheet within the hydrogel. These localized stress points may have caused hydrogel rupture under uniform force. Based on strain−stress analysis, GelMA/BP50 was identified as the optimal hydrogel for in vitro and in vivo desensitizing therapy.

The swelling properties of elastic hydrogel are governed by water absorption driven by capillary force, with swelling influenced by factors, such as pH, temperature, and porous structure of the hydrogel.^[^ ^24^ ^]^ Increased cross‐linking density typically results in a lower swelling ratio. As shown in Figure 2I, the swelling ratio of GelMA/BP decreased from 36.5% to 32.5% as BP nanosheet concentration increased, likely due to the reduced pore size and lower water absorption capacity. Photothermal studies demonstrated a rapid increase in temperature within the first 2 min of laser irradiation, followed by gradual stabilization (Figure 2J,K). The final temperatures of GelMA/BP50 and GelMA/BP100 groups reached or exceeded 50 °C, while the GelMA/BP25 group exhibited a significantly lower temperature that remained unchanged with extended irradiation time. Biodegradability is another crucial characteristic of hydrogels, particularly in drug release applications. Degradation experiments simulating the oral microenvironments using artificial saliva and collagenase II showed that GelMA/BP degraded faster than GelMA alone. However, the degradation rate was not significantly affected by BP nanosheet concentration (Figure 2L). Additionally, inductively coupled plasma mass spectrometry results indicated that GelMA/BP50 released 0.02 mg mL^−1^ of P^−^ within 48 h (Figure 2M), supporting the potential for controlled BP nanosheet degradation in an orthotopic DH model. The combination of efficient photothermal conversion, continuous P^−^ release, and Ca^2+^ recruitment suggested GelMA/BP had a strong potential for efficiently occluding dentinal tubules during DH therapy.

### 2.3 Desensitizing Efficacy of Black Phosphorus Nanosheet‐Composited Methacrylate Gelatin Hydrogel in vitro

**Figure 3:**
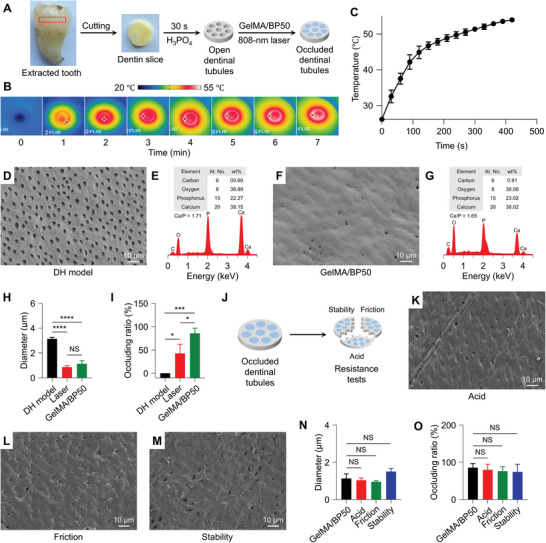
Desensitizing efficacy of GelMA/BP in vitro. A) DH model construction. B,C) Infrared images (B) and temperature curve (C) of dentin surface. D) FE‐SEM image of exposed dentinal tubules in DH model. E) EDX spectrum of dentin surface after being etched. F) FE‐SEM image of dentin surface in GelMA/BP50 group. G) EDX spectrum of dentin surface in GelMA/BP50 group. H,I) Diameters (H) and occluding ratios (I) of dentinal tubules on dentin surface. J) Resistance tests. K−M) FE‐SEM results of dentin slices after acid challenge (K), friction challenge (L), and stability challenge experiments (M). N,O) Diameters (N) and occluding ratios (O) of dentinal tubules on dentin surface after resistance tests. All statistical data are represented as mean ± SD (*n* = 3; NS, No significant difference; ^*^ *P* < 0.05, ^**^ *P* < 0.01, ^***^ *P* < 0.001, and ^****^ *P* < 0.0001).

A typical feature of DH is the presence of exposed dentinal tubules on the dentin surface. As depicted in **Figure** **3**A, an exposed dentinal tubule model was constructed through mechanical cutting and phosphoric acid (H_3_PO_4_) etching, followed by PTT on dentin slices with GelMA/BP50. The temperature change on the dentin surface was monitored (Figure 3B,C). The dentin surface temperature increased by 27.8 °C, underscoring the potent photothermal properties of GelMA/BP50.

Following the construction of DH model, FE‐SEM imaging revealed a multitude of open dentinal tubules on the dentin surface (Figure 3D), with a Ca/P ratio of 1.71 (Figure 3E).^[^ ^25^ ^]^ A single laser treatment reduced the diameters of most dentinal tubules but did not completely seal them with a Ca/P ratio of 2.22 (Figure S3A,B, Supporting Information). The increased Ca/P ratio is attributed to the evaporation of organic components under laser irradiation conditions.^[^ ^25^ ^]^ Simultaneously, under acidic, abrasive, and salivary conditions, many dentinal tubules are re‐opened (Figure S3C−E, Supporting Information), sparking interest in combining laser therapy with desensitizing agents to enhance tubule occlusion. The FE‐SEM images of GelMA/BP50‐treated group revealed that most dentin tubules were effectively occluded, with a significant reduction in diameters of the remaining unobstructed dentinal tubules (Figure 3F). The dentin surface Ca/P ratio in this group was 1.65 (Figure 3G).

Under physiological conditions, dental demineralization and remineralization occur in a dynamic equilibrium. Excessive demineralization leads to the degradation of dental hard tissue and collagen. The DH model promotes demineralization, opening the dentinal tubules.^[^ ^26^ ^]^ The Ca/P molar ratio, used to evaluate the solubility of calcium phosphate compounds on the tooth surface, was measured via energy dispersive X‐ray (EDX). HA, the primary component of dentin, typically has a Ca/P ratio of 1.67.^[^ ^27^ ^]^ Demineralization or acid etching increases this ratio. Following co‐culture dentin slices with saliva for seven days, the Ca/P ratio approached 1.67, suggesting that HA may be the main mineral component on the dentin surface.^[^ ^28^ ^]^ GelMA/BP50, as a biomineralization platform, underwent continuous degradation under laser irradiation and in the presence of saliva, releasing P^−^ into dentinal tubules. Simultaneously, GelMA/BP50 facilitated the diffusion of Ca^2+^ from the surrounding microenvironments into the dentin, promoting remineralization.

Using ImageJ software, the occlusion ratio and dentinal tubule diameter were quantified following laser treatment. The etched dentin surface exhibited dentinal tubules with diameters ranging from 0 to 3.14 µm. A single laser treatment reduced the diameter to 0−0.87 µm, achieving an occluding rate of 43%. When GelMA/BP50 was applied during laser irradiation, the tubule diameter was further reduced to 0−1.13 µm, increasing the occlusion rate to 86% (Figure 3H,I). While the laser treatment alone demonstrated a more significant reduction in dentinal tubule diameter, the overall occlusion efficacy of GelMA/BP50 was notably superior. During the desensitization process, GelMA/BP50 converted light energy into heat, generating a stable photothermal effect that reduced tubule diameter.

Factors, such as acidic dietary intake and frictional forces, influence desensitization efficiency. Most commercially available desensitizers display limited acid and friction resistance, as well as poor stability after prolonged exposure to saliva.^[^ ^29^ ^]^ To evaluate acid resistance, abrasion resistance, and desensitizing stability, dentin samples were partitioned into three groups for resistance tests (Figure 3J). No significant differences were observed in diameters or occlusion rates of the dentinal tubules after testing compared to pre‐test conditions (Figure 3K−O). These findings indicated that GelMA/BP50 treatment exhibited strong acid resistance (80% occlusion rate), friction resistance (76% occlusion rate), and long‐term stability (74% occlusion rate), providing valuable insights for clinical applications. GelMA/BP50 exhibited a synergistic desensitization mechanism, combining dentinal tubule occlusion via a photothermal effect with enhanced dentin biomineralization through ion precipitation.

### 2.4 Desensitizing Efficacy of Black Phosphorus Nanosheet‐Composited Methacrylate Gelatin Hydrogel in vivo

**Figure 4:**
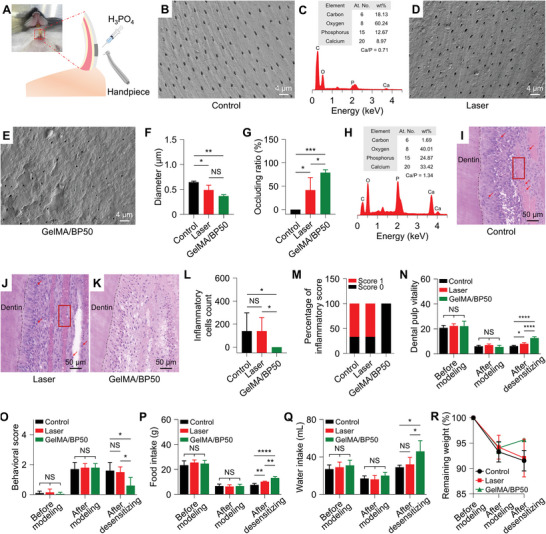
Desensitizing efficacy of GelMA/BP in vivo. A) Rat DH model construction. B) FE‐SEM image of Control group. C) EDX spectrum of dentin surface after etching. D,E) FE‐SEM images of Laser group (D) and GelMA/BP50 group (E). F,G) Diameters (F) and occluding ratios (G) of dentinal tubules. H) EDX spectrum of dentin surface after treatment with GelMA/BP50 hydrogel under laser irradiation. I−K) H&E staining results of dental pulp response of Control group (I), Laser group (J), and GelMA/BP50 group (K). The red box indicates dental pulp hyperemia. L,M) Inflammatory cells count (L) and a percentage of an inflammatory score (M) of dental pulp. N) Results of dental pulp vitality test of SD rats before modeling, after modeling, and after desensitizing. O) Behavioral scores of SD rats before modeling, after modeling, and after desensitizing. P,Q) Food intake (P) and water intake (Q) of SD rats before modeling, after modeling, and after desensitizing. R) Residual weight of SD rats after modeling and desensitizing. All statistical data are represented as mean ± SD (*n* = 5; NS, No significant difference; ^*^ *P* < 0.05, ^**^ *P* < 0.01, ^***^ *P* < 0.001, and ^****^ *P* < 0.0001).

While in vitro experiments demonstrated a significant desensitization effect, they were limited in replicating the complex oral microenvironments. To address this limitation, an in vivo rat DH model was utilized to evaluate the desensitizing efficacy of GelMA/BP50. The rat model of DH was established using a high‐speed air turbine handpiece and H_3_PO_4_ to compare the desensitizing efficacy of the Control, Laser, and GelMA/BP50 groups (**Figure** **4**A). FE‐SEM revealed extensive dentinal tubules on the dentin surface of Control group (Figure 4B), with a Ca/P ratio of 0.71 (Figure 4C). The observed decrease in this ratio may be attributed to the influence of proteins or peptides in oral saliva and the exudation of organic matter within the dental pulp.^[^ ^30^ ^]^ Following desensitization treatment, both the GelMA/BP50 (0−0.37 µm) and Laser groups (0−0.49 µm) exhibited significant reductions in the diameters of dentinal tubules compared to the Control group (0−0.65 µm) (Figure 4D−F). However, GelMA/BP50 treatment achieved a significantly higher occlusion ratio (79%) than the Laser group (42%) (Figure 4G). Temperature changes on the rat dentin surface were also monitored during desensitization (Figure S4, Supporting Information). The GelMA/BP50 group exhibited a significant temperature increase, exceeding 40 °C after 1 min of laser irradiation, and the hydrogel maintained its photothermal properties in the oral microenvironments throughout the experiment. After desensitization, the Ca/P ratio of dentin surface in the GelMA/BP50 group reached 1.34 (Figure 4H), comparable to the low Ca/P ratio reported for rat teeth.^[^ ^31^ ^]^ This lower ratio may be due to the partially substituting Ca^2+^ with magnesium during mineralization. The elevated levels of Ca^2+^ and P^−^ in the GelMA/BP50 group suggested promising mineralization potential. In vivo, desensitizing therapy further demonstrated the potential of GelMA/BP50 for DH management. However, the degree of dentinal tubule occlusion achieved in vivo was slightly lower than that observed in vitro, likely due to the influence of saliva, oral flora, and diet in the rat model. It is also important to note that the oral microenvironments of rats differ significantly from humans, so further clinical studies are needed to validate the desensitization efficacy of this hydrogel in humans.

Dental pulp tissue is crucial for nourishing, sensing, and protecting the tooth.^[^ ^32^ ^]^ Stimulation by external factors induces vascular congestion within the pulp, which has limited collateral circulation.^[^ ^33^ ^]^ Prolonged stimulation leads to irreversible pulp damage. To assess the impact of photothermal desensitization on dental pulp tissue, hematoxylin and eosin (H&E) staining, pulp vitality testing, and behavioral assessments of rats were conducted before modeling, after modeling, and after desensitizing. In individuals with DH, open dentinal tubules allow bacteria and food debris to penetrate, potentially irritating the dental pulp and causing hyperemia or inflammation.^[^ ^34^ ^]^ H&E staining revealed the presence of inflammatory cells and red blood cells (RBCs) in the dental pulp tissue of the Control and Laser groups (Figure 4I,J). In contrast, the GelMA/BP50‐treated pulp exhibited no significant RBC exudation or inflammatory cells, and odontoblasts maintained a typical pseudostratified morphology (Figure 4K). Detailed results of H&E staining are provided in Supporting Information (Figure S5, Supporting Information), which showed numerous inflammatory cells in pulpitis tissue. The GelMA/BP50 group had significantly fewer inflammatory cell counts and lower inflammation scores than the Control and Laser groups (Figure 4L,M), suggesting that GelMA/BP50 effectively mitigated the stimulatory external stimulation of dental pulp.

Brännström's hydrodynamic theory posits that fluid movement within dentinal tubules contributes to toothache in patients with DH.^[^ ^35^, ^36^ ^]^ Damage to the enamel layer by external factors increases fluid flow in the dentinal tubules, even in response to minimal electrical stimulation, triggering contraction of odontoblasts and stimulating pulpal nerves, resulting in toothache. Pulp vitality tests are essential in evaluating tubule occlusion and the condition of dental pulp. Prior to modeling, all rats' teeth had vital dental pulp tissue. After modeling, exposed dentinal tubules reduced overall pulp vitality, as tiny electrical stimulation increased fluid flow in the tubules. Effective desensitization treatments, like GelMA/BP50, were associated with increased pulp vitality test values (Figure 4N). Cold stimulation is another key factor in assessing desensitization efficacy. Intact teeth are resistant to cold stimuli, but after acid etching, cold‐induced toothache significantly increased behavioral scores. After desensitization treatment with GelMA/BP50, the behavioral scores were significantly lower compared to the other groups (Figure 4O). In animals with DH, transient food residue and water cause transient stimulation, leading to toothache and changes in food and water intake, consequently resulting in alterations in their body weight.^[^ ^37^ ^]^ The GelMA/BP50 group exhibited a 97.6% increase in food intake compared to pre‐treatment levels, while the Laser group demonstrated a 59.2% increase, and the Control group showed a 13.7% increase (Figure 4P). Water intake increased significantly across all groups, with the GelMA/BP50, Laser, and Control groups showing 130.0%, 100.0%, and 70.6%, respectively (Figure 4Q). Chronic irritation from food debris block dentinal tubules, leading to persistent pulp irritation and inflammation, which may explain the more pronounced differences in food intake compared to water intake among the groups. The changes in dietary intake further impacted body weight: the Laser group lost weight after treatment, whereas the GelMA/BP50 group showed a significant increase in body weight compared to the pre‐treatment levels (Figure 4R). Effective desensitization treatment by occluding dentinal tubules isolates the pulp tissue from irritants, such as food and water, alleviating toothache and promoting weight gain.

## 3 Conclusion

A unique GelMA/BP was developed as a dual‐function platform, combining PTT and biomineralization, to treat DH. Desensitization treatment with GelMA/BP50 reduced dentinal tubule diameter to 0−1.13 µm with an occluding ratio of 86% in vitro. In vivo, GelMA/BP50 exhibited similar efficacy, with an 79% occlusion ratio with a reduced dentinal tubule diameter of 0−0.37 µm. GelMA/BP utilized photothermal conversion to melt the superficial dentin, effectively reducing dentinal tubule diameters while promoting dentin biomineralization by facilitating the penetration of P^−^ and introducing Ca^2+^ within dentinal tubules, leading to the formation of HA analogs. The dual‐action mechanism of GelMA/BP effectively occluded dentinal tubules in the DH model, demonstrating stable closure both in vitro and in vivo while significantly alleviating toothache symptoms. GelMA/BP shows promise as a future effective treatment for DH due to its non‐invasive, long‐lasting effects, and potential to enhance dental tissue repair, warranting further research for clinical application.

## Conflict of Interest

The authors declare no conflict of interest.

## Supporting information



Supporting Information

## Data Availability

The data that support the findings of this study are available from the corresponding author upon reasonable request.
