# Supplementary material for: Photothermal Effect and Biomineralization of Black Phosphorus Nanosheet‐Composited Hydrogel Boosts Synergistic Treatment of Dentin Hypersensitivity
Source: Adv Sci (Weinh). 2025 Jan 3;12(9):2412561. doi: 10.1002/advs.202412561 (PMC11884576; doi:10.1002/advs.202412561)
Supplement: Supplementary file 1 — Supporting Information [file ADVS-12-2412561-s001.docx]

Supporting Information

**Photothermal Effect and Biomineralization of Black Phosphorus Nanosheet-Composited Hydrogel Boosts Synergistic Treatment of** **Dentin Hypersensitivity**

*Qihui Wang, Guoliang Wang, Xinru Li, Di Li, Congxiao Zhang,* and Jianxun Ding**

Q. Wang, X. Li, Prof. C. Zhang

Department of Stomatology, The First Hospital of Jilin University

1 Xinmin Street, Changchun 130061, P. R. China

E-mail: zhangcongxiao@jlu.edu.cn

Q. Wang, X. Li, Prof. G. Wang, Prof. J. Ding

Key Laboratory of Polymer Ecomaterials

Changchun Institute of Applied Chemistry, Chinese Academy of Sciences

5625 Renmin Street, Changchun 130022, P. R. China

E-mail: jxding@ciac.ac.cn

Prof. D. Li

Department of Hepatobiliary and Pancreatic Surgery, General Surgery Center, The First Hospital of Jilin University

1 Xinmin Street, Changchun 130061, P. R. China

Prof. J. Ding

School of Applied Chemistry and Engineering

University of Science and Technology of China

96 Jinzhai Road, Hefei 230026, P. R. China

**Experiment Section**

*Materials:* Black phosphorus (BP) was purchased from MKNANO^®^ Tech. Co., Ltd. (MK903, Nanjing, P. R. China). 1-Methyl-2-pyrrolidinone (NMP) was obtained from Aladdin Scientific Corp (M100589, Shanghai, P. R. China). Dulbecco's phosphate-buffered saline (DPBS) was purchased from Gibco™ (14190-144, Billings, MT, United States). Methacrylate gelatin (GelMA) was purchased from Aladdin Scientific Corp. (M299513, Shanghai, P. R. China). Lithium phenyl-2,4,6-trimethylbenzoylphosphinate (LAP) was supplied by Yuanye Bio-Technology Co., Ltd. (Y43995, Shanghai, P. R. China). Collagenase II was purchased from Solarbio^®^ (C8150, Beijing, P. R. China). Fusayama was purchased from Yuanmu Bio-Technology Co., Ltd. (R18795, Shanghai, P. R. China). Kimtech Science Kimwipes were purchased from Kimberly·Clark Inc. (34155, Dallas, TX, United States). Ketamine was purchased from Sinopharm Chemical Reagent Co., Ltd. (Beijing, P. R. China).

*Preparation and Characterizations of BP Nanosheet:* BP nanosheet was synthesized by liquid exfoliation of BP powder. After grinding in a mortar, BP was suspended in NMP and sonicated with a sonic tip for 20 h under continuous cooling (sonication power: 30%, on/off cycle: 3 s/3 s) using an Ultrasonic homogenizer (JY 92-IIN, Ningbo Scientz Biotechnology Co., Ltd., Ningbo, P. R. China). The dispersion was centrifuged at 3500 rpm for 10 min to remove bulky BP powder, followed by centrifugation at 9000 rpm for 10 min to isolate BP nanosheet. Then, the BP nanosheet was redispersed in deionized water for further use. A diluted BP suspension (25.0 µg mL^−1^) was dropped onto oblique aluminum foil. After drying, the morphology of BP nanosheet was then recorded by field emission scanning electron microscope (FE-SEM; Merlin VP compact, Carl Zeiss, Jena, Germany). BP suspension (25.0 µg mL^−1^) was dropped onto the copper mesh and dried overnight, and the morphology of BP was recorded by transmission electron microscope (TEM; JEOL Ltd., Tokyo, Japan). The BP (25.0 µg mL^−1^) size was measured by dynamic light scattering (DLS; Malvern Panalytical Zetasizer Nano-ZS; Malvern Panalytical Ltd., Malvern, UK) (*n* = 3). All statistical data are represented as mean ± standard deviation (SD; SPSS Statistics). Raman scattering spectra of BP suspension (1.0 mg mL^−1^) were measured using a high-resolution confocal Raman microscope (Horiba LabRAM HR Evolution Raman microscope, Horiba Jobin Yvon, Kyoto, Japan) with 532 nm laser excitation (*n* = 3), and data were analyzed using Origin software.

*Preparation and Characterizations of GelMA and GelMA/BP Hydrogel:* The GelMA sponge was dissolved in DPBS (1×) containing 0.1% (*W/V)* LAP at 60 °C for 20 min with continuous oscillation to ensure complete dissolution. Subsequently, 10% and 15% (*W/V)* GelMA hydrogels were prepared by photo-cross-linking under 405 nm blue light (ANJOET) irradiation for 90 s. The cylindrical GelMA hydrogels (high: 3 mm, diameter: 10 mm) were implemented in rheology experiments, while compression tests were conducted on cylindrical hydrogels (high: 6 mm, diameter: 10 mm) using a universal testing machine at 37 °C. Based on those mechanical results, 15% (*W/V)* GelMA was selected for forming GelMA/BP. BP suspension was rinsed three times with deionized water to remove residual NMP. BP dispersions at varying concentrations were sonicated with GelMA prepolymer solution in a 1:1 ratio for 30 min. Resulting GelMA/BP solutions were then exposed to 405 nm blue light for 90 s to form hydrogels. GelMA/BP was prepared with BP nanosheet concentrations of 25.0, 50.0, and 100.0 µg mL^−1^, and designated as GelMA/BP25, GelMA/BP50, and GelMA/BP100, respectively. After freeze-drying, GelMA and GelMA/BP hydrogels were sputter-coated with gold, and their porous morphology was observed using FE-SEM. The size of the porous hydrogel by Nano Measurer was calculated (Department of Chemistry, Fudan University, P. R. China). To illustrate the successful cross-linking of GelMA and BP, GelMA/BP was examined on a high-resolution confocal Raman microscope with a 532 nm laser excitation (*n* = 3). To test the mechanical properties of GelMA/BP, cylindrical hydrogels were implemented in rheology experiments (hydrogel high: 3 mm, diameter: 10 mm) on a rheometer operating and compression experiments (hydrogel high: 6 mm, diameter: 10 mm) on a universal testing machine at 37 °C.

The swelling behavior of hydrogel was calculated by equilibrium swelling ratio (SR) according to Eq. (1). Briefly, freeze-dried hydrogel disks were weighed (*W_d_*) and then immersed in DPBS at 37 °C for 24 h. Subsequently, hydrogels were removed and wiped with Kimwipes to remove excess liquid, and the swollen weight (*W*_s_) was recorded (*n* = 3).

$$SR=\frac{W_{s}-W_{d}}{W_{d}}\times100\% (1)$$

To evaluate the degradation rate, GelMA and GelMA/BP were incubated in artificial saliva with collagenase II (1.5 U mL^−1^) on an orbital shaker (100 rpm) at 37 °C (*n* = 3). At predetermined intervals, hydrogels were freeze-dried and weighted to calculate the percentage of remaining weight after enzymatic degradation. Cylindrical GelMA/BP50 (high: 6 mm, diameter: 10 mm) was soaked in deionized water in glass vials and exposed to 808 nm laser irradiation for 5 min every 12 h. The solution was sampled every 12 h from each glass vial to determine the amount of released phosphorus using inductively coupled plasma mass spectrometry (ICP-MS; Agilent 7800, Santa Clara, CA, United States) (*n* = 3).

*Near-Infrared Laser-Induced Heat Conversion:* 1.0 mL of aqueous solutions of BP nanosheet (6.25, 12.5, 25.0, 50.0, and 100.0 µg mL^−1^) were irradiated with near-infrared (NIR) laser (808 nm laser, DS3-11313-0411, BWT Beijing Ltd., Beijing, P. R. China) at 1.0 W cm^−2^ for 10 min (*n* = 3). The laser was positioned 3 cm above the BP suspension. The temperature of BP solutions was recorded every 30 s with an infrared thermal imaging camera (FLIR One) to monitor the influence of different concentrations on the photothermal effect. In this process, the camera remained parallel to the BP suspension. BP suspension (25.0 µg mL^−1^) was irradiated with varying 808nm laser powers (0.5, 0.75, 1.0, 1.25, and 1.5 W cm^−2^) for 10 min to measure the influence of laser power on the photothermal effect (*n* = 3), with temperatures recorded every 30 s. Similarly, GelMA/BP hydrogels were irradiated with the 808 nm laser at 1.0 W cm^−2^ for 10 min, and the temperature of hydrogel was recorded every 30 s to examine the effect of the addition of GelMA on BP's photothermal effect (*n* = 3).

The photothermal stability of BP nanosheet (25.0 µg mL^−1^) was assessed through four on/off cycles of laser irradiation. The photothermal conversion efficiency (*η*) of BP nanosheet was calculated according to the following formula Eq. (2)−(5):

$$\eta=\frac{hS\left( T_{\max}-T_{\mathrm{surr}} \right)-Q_{0}}{I\left( 1-{10}^{-A_{808}} \right)}\cdots\cdots\left( 2 \right)$$

$$\tau_{s}=\frac{m_{d}C_{d}}{hS}\cdots\cdots\left( 3 \right)$$

$$\theta=\frac{T-T_{\mathrm{surr}}}{\left( T_{\max}-T_{\mathrm{surr}} \right)}\cdots\cdots\left( 4 \right)$$

$$Q_{0}=hS\left( T_{max, water}-T_{\mathrm{surr}} \right)\cdots\cdots\left( 5 \right)$$

The thermal time constant (*τ*_s_ = 341.56815) was derived from the linear regression curve in the cooling phase of BP nanosheet (25.0 µg mL^−1^) irradiated at 1.0 W cm^−2^. The *m*_d_ and *C*_d_ represented the mass of solution (greater than 1 g) and heat capacity (4.2 J g^−1^ K^−1^), respectively, from which the value of *hS* was calculated. *Q*_0_ was determined *via* Eq. (5), which indicated the background energy input in the absence of a BP nanosheet. *T*_max, water_ and *T*_surr_ represented the steady-state maximum temperature of water (29.9 °C) and the ambient room temperature (28.7 °C), respectively. Once *hS* and *Q*_0_ were determined, *η* (79.4%) could be calculated based on Eq. (2). *T*_max_ indicated the steady maximum temperature of BP nanosheet solution (53.9 °C). *I* and *A*_808_ represented the laser power (1.0 W cm^−2^) and absorbance of BP at 808 nm (0.202), respectively.

*Preparation of Dentin Slices in vitro:* In total, nine impacted mature human third molars with no caries were collected with ethical approval from the First Hospital of Jilin University (23K055-001) and patients' informed consent. All donated patients signed informed consent forms and authorized us to use their extracted teeth for this experiment. The middle crown area of teeth was sectioned using a hard tissue cutting machine (SYJ-150, P. R. China) to obtain uniform 2 mm thick dentin slices. Then, the dentin slices were polished sequentially with #400, #1000, #1500, and #2000 silicon carbide sandpaper. The dentin slices were then ultrasonically washed in deionized water for 2 min to remove debris and dried with Kimwipes. Subsequently, the dentin slices were treated with 35% phosphoric acid for 2 min to open dentinal tubules, followed by washing with deionized water to remove any residual acid. Finally, the dentin slices were stored in deionized water at 4 °C for later use.

*Efficacy of GelMA/BP50 in Occlusion of Dentinal Tubules in vitro:* Dentin slices were randomly divided into three groups: dentin hypersensitivity (DH)-model group, laser group, and GelMA/BP50 group (*n* = 3). The open dentinal tubules and Ca/P ratio of dentin surface were detected by FE-SEM and energy dispersive X-ray (EDX; EDAX Genesis 2000, AMETEK, Wiesbaden, Germany) after the DH model was established. FE-SEM assessed the diameter range of dentinal tubules. The weight percentages (wt%) of Ca and P on the dentin slices were determined by EDX, and the Ca/P ratio of the dentin surface was calculated from their weight percentage. The laser group was treated with single NIR laser irradiation (1.0 W cm^−2^) for 7 min, with the laser positioned 3 cm above the dentin surface. Pre-prepared GelMA/BP50 solution was dropped on the dentin surface and cross-linked with 405-nm blue light. Subsequently, dentin slices were subjected to laser irradiation, and an infrared thermal imaging camera recorded the photothermal effect on the dentin surface in the GelMA/BP50 group. After desensitizing, the dentin slices were observed with FE-SEM to assess the occlusion of dentinal tubules. To evaluate resistance to external stimuli, dentin slices treated with GelMA/BP50 were divided into three parts for tests. Acid resistance was evaluated by soaking slices in 35% (*V*/*V*) phosphoric acid for 30 s. Friction resistance was assessed by treating the slices with an electric toothbrush (Royalstar, RSD-YSA1S2) for 30 s. Stability was tested by soaking the dentin slices in artificial saliva at 37 °C for seven days, with the artificial saliva being refreshed every 24 h to mimic the flow of natural saliva. After these treatments, the occlusion of dentinal tubules was observed by FE-SEM. The diameter change and occluding ratio of dentinal tubules were analyzed with ImageJ (National Institutes of Health, Rockville, MD, United States; http://imagej. net/ImageJ) and calculated by Eq. (6). EDX detected mineralization growth on the dentin surface after the dentin slices were washed and dried following their storage in artificial saliva.

$$Occluding ratio=\left( \frac{\begin{aligned} Numbers of complete \\ occluded dentinal tubules \end{aligned}}{Numbers of total dentinal tubules} \right)\times100\%\cdots\cdots(6)$$

*Surgical and Desensitizing Procedures in vivo:* Fifteen Sprague-Dawley (SD) male rats (seven weeks, Changsheng Bio-technology Co., Ltd, Liaoning, P. R. China) were used in this study with approval from the Ethical Committee (Changchun Institute of Applied Chemistry, Chinese Academy of Sciences, IACUC Issue NO. CIAC2022[0094]). Rats were anesthetized using ketamine with intraperitoneal injection, and teeth cavities (length: 3 mm, width: 2 mm, depth: 1 mm) were prepared on the labial surface of the incisors (both two incisors of rats) using a high-speed air turbine handpiece (Liangya Dental Equipment, P. R. China). Exposed dentin cavities were etched with 35% (*V*/*V*) phosphoric acid for 30 s to remove the smear layer and open dentinal tubules to simulate DH, followed by thorough washing with deionized water. One day later, animals were randomly divided into three groups: Control group, Laser group, and GelMA/BP50 group (*n* = 5). The Control group received no treatment. The Laser group was irradiated with a single NIR laser (1.0 W cm^−2^) for 7 min at a distance of 3 cm above the dentin surface. In the GelMA/BP50 group, the pre-prepared hydrogel prepolymer was applied to the dentin surface and photo-cross-linked, followed by NIR laser irradiation at 3 cm above the dentin surface for 7 min. Temperature change on the dentin surface was recorded every 1 min during desensitizing. Incisors of rats were extracted one day after desensitization (Left incisor). Then, the efficacy of dentinal tubule occlusion was analyzed by FE-SEM. EDX analyzed the Ca/P ratio of the rat dentin surface. ImageJ calculated diameter changes and occluding ratio of dentinal tubules.

*Dental Pulp Response in vivo:* Incisors of rats were extracted one day after desensitization (Right incisor). The extracted dental specimens were prepared for histological analysis using hematoxylin and eosin (H&E) staining to preliminary judge dental pulp inflammation response. The H&E staining procedure included the following steps: 1) Fixation: Tissues are fixed with formaldehyde; 2) Dehydration and Clearing: Tissues are dehydrated with alcohol and cleared with xylene; 3) Impregnation: Tissues are embedded in paraffin wax; 4) Sectioning: Thin sections are cut and mounted on slides; 5) De-waxing: Wax is removed with xylene, followed by rehydration; 6) Staining: Sections are stained with hematoxylin (nuclei) and eosin (cytoplasm); 7) Dehydration, Clearing, and Mounting: Sections are dehydrated, cleared, and cover-slipped; 8) Microscopic Examination: Slides are examined under a microscope.

Inflammatory cell counts and inflammation scores on dental pulp tissue were assessed based on H&E images of the dental pulp tissue. Inflammatory cells, characterized by a distinctive morphology featuring an enlarged, irregular shape and lobulated nucleus, were counted in 10 randomly selected regions, representing 1/100 of the target image area, repeated thrice. Inflammation scores were assigned based on the following criteria: 0-no inflammation, 1-mild inflammation with a few inflammatory cells and exudated RBCs, 2-moderate inflammation with inflammatory cells invading the odontocytic-dentin area (odontoblasts pseudo-lamellar appearance not be recognized), 3-severe inflammation with obvious cellular infiltration, including abscess formation.^[1]^ The dental pulp vitality test and behavioral score were recorded to judge dental pulp response to external stimuli before modeling, after modeling, and after desensitization. The dental pulp vitality test was performed using a pulp tester (Jinyuan E-Commerce Technology Co., Ltd., Foshan, P. R. China) applied vertically to the exposed dentin. Responses, such as soreness, numbness, and toothache, experienced by rats within the range of 0−40 indicated vital dental pulp, while a response observed in the range of 40−80 suggested partial necrosis. A lack of response when the value reached 80 signified that the dental pulp was necrotic. For behavioral scoring, animals were fastened to a stationary frame, and cold water was applied to the exposed dentin for 5 s to observe their physical reactions. Behavioral scores include 0-no response, 0.5-slight body contraction, 1-strong body contraction, 2-strong body contraction with short vocalization, and 3-strong body contraction with prolonged vocalization.^[2]^ Two experimenters independently performed the tests simultaneously, calculating the average values. Rats were provided with weighed food and water daily, and their food consumption (g) and water intake (mL) were recorded to calculate daily variations. The body weight (g) of each rat was also monitored. Changes in food intake, water intake, and body weight were assessed as indicators of desensitizing efficacy before modeling, after modeling, and after desensitizing.

*Statistical Analysis*: Data are represented as mean ± SD. Statistical analyses were carried out using GraphPad Prism software. The statistical significance of the differences between groups was determined using Student's *t*-test or one-way ANOVA. Differences were considered statistically significant at **P* <0.05, ***P* < 0.01, ****P* < 0.001, *****P* < 0.0001. NS was defined as no significant difference.

**References**

[1] Y. Chiang, H. Lin, H. Chang, Y. Cheng, H. Tang, W. Yen, P. Lin, K. Chang, C. Lin, *ACS Nano* **2014**, *8*, 12502.

[2] M. R. Bergamini, M. M. Bernardi, I. B. Sufredini, M. T. Ciaramicoli, R. M. Kodama, F. Kabadayan, C. H. C. Saraceni, *Life Sci.* **2014**, *98*, 96.


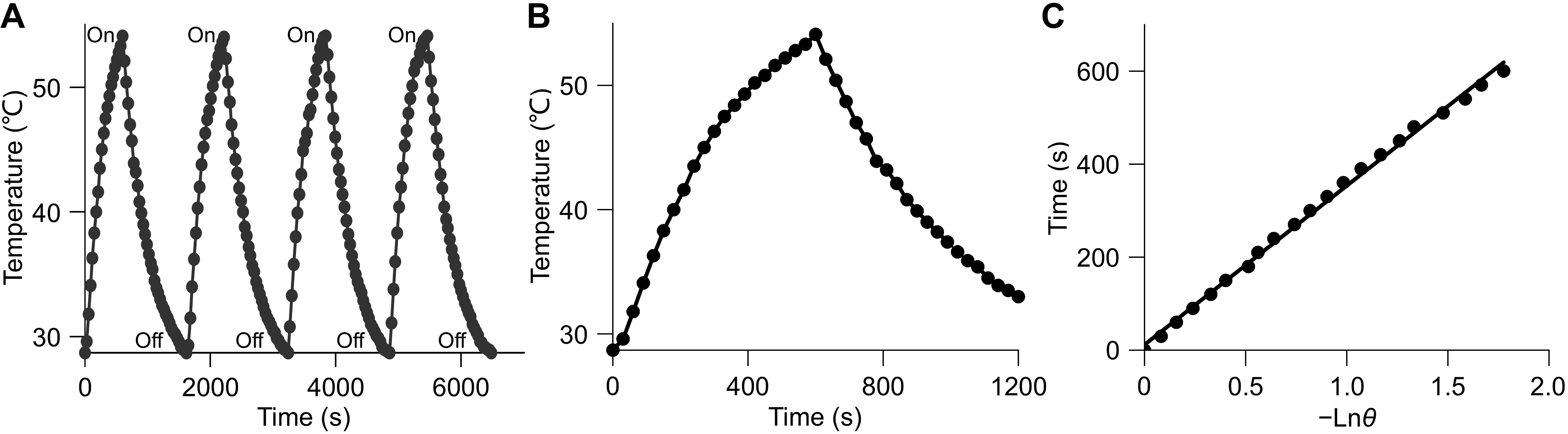


**Figure S1.** Temperature curves of 25.0 μg mL^−1^ BP suspension. A) Temperature cycle curve, B) rising-decreasing temperature curve, and C) photothermal fitting curve of 25.0 μg mL^−1^ BP suspension.

**
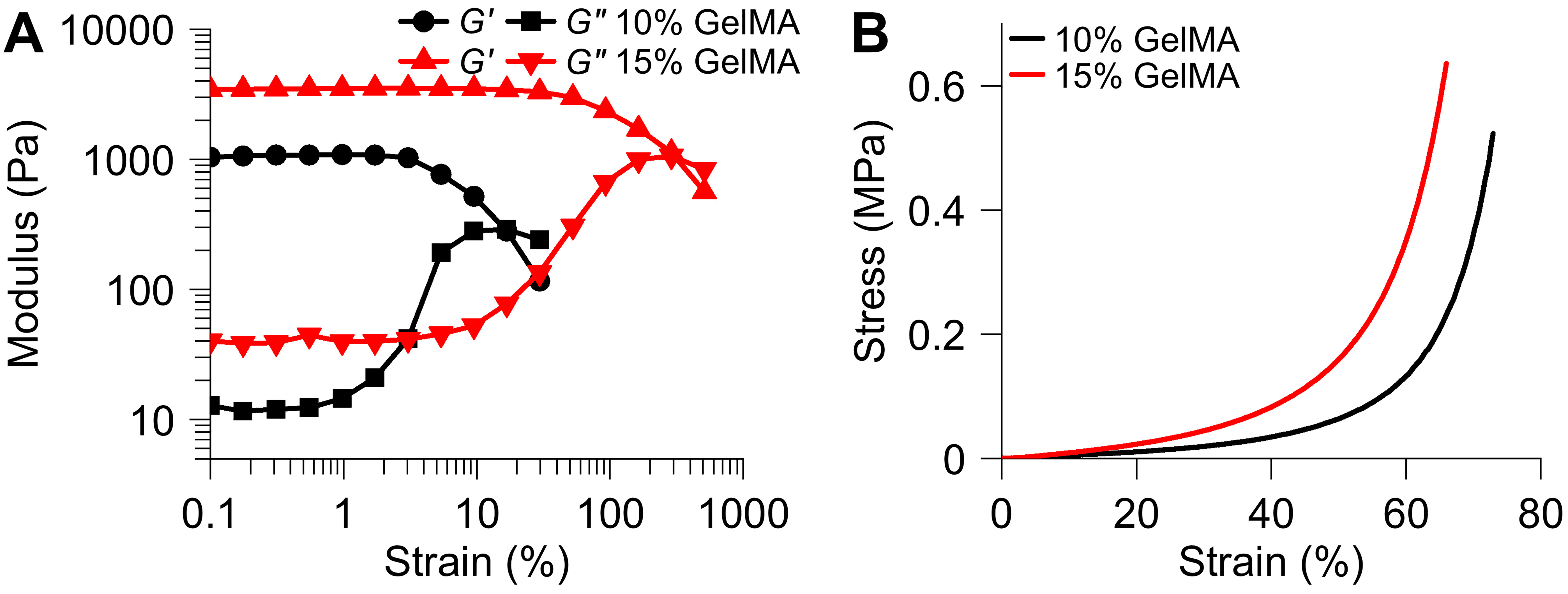
**

**Figure S2.** Mechanical properties of GelMA hydrogels. A) Amplitude scanning strain curve and B) strain−stress curve of 10% and 15% (*W/V*) GelMA hydrogels.


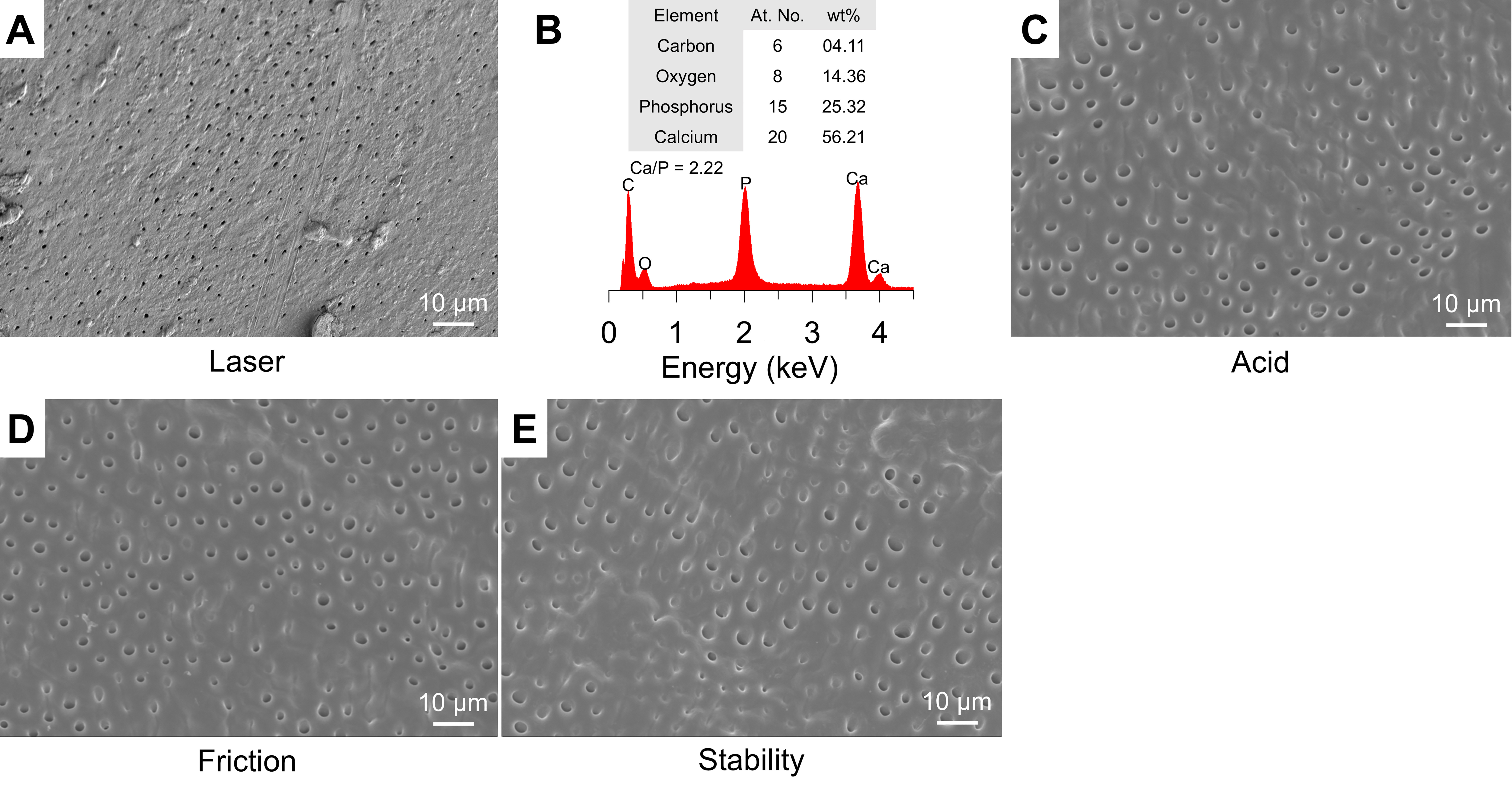


**Figure S3**. Dentin surface after desensitizing with a single laser irradiation. A) FE-SEM image of dentin surface after treatment with a single laser. B) EDX spectrum of dentin surface in Laser group. C) FE-SEM results of dentin slices after acid challenge, D) friction challenge, and E) stability challenge experiments.


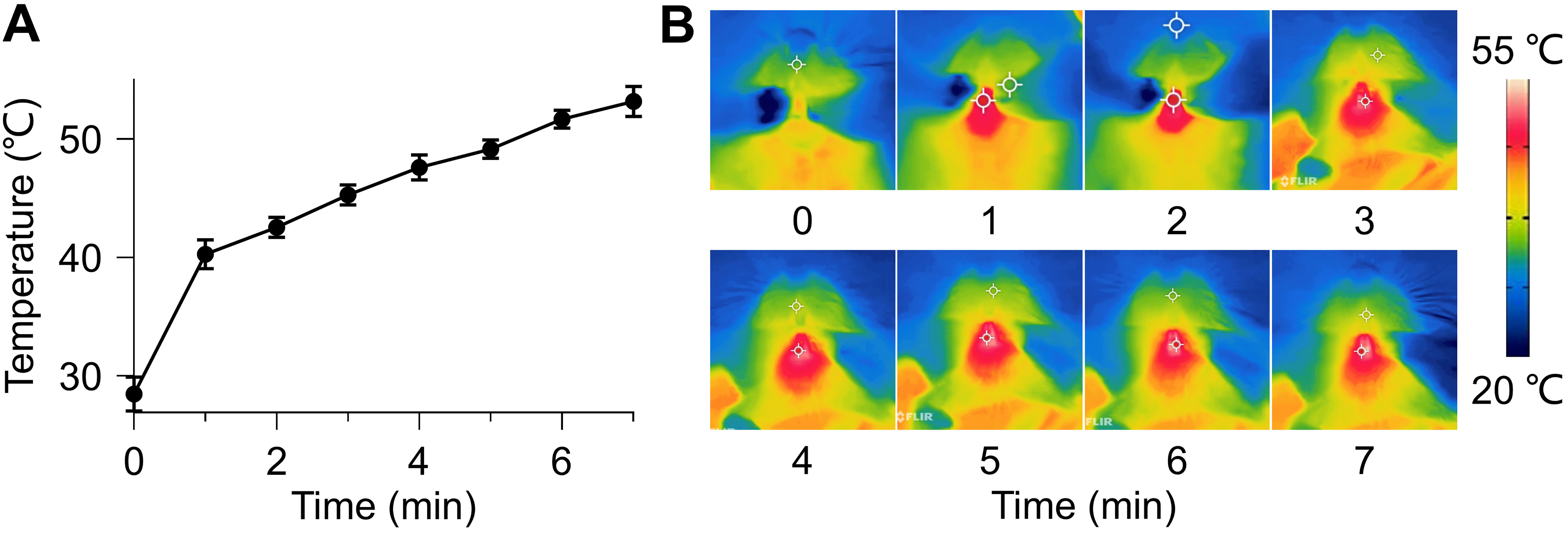


**Figure S4.** Photothermal tests of rat dentin surface. A) Temperature curve and B) infrared images of rat dentin surface.

**
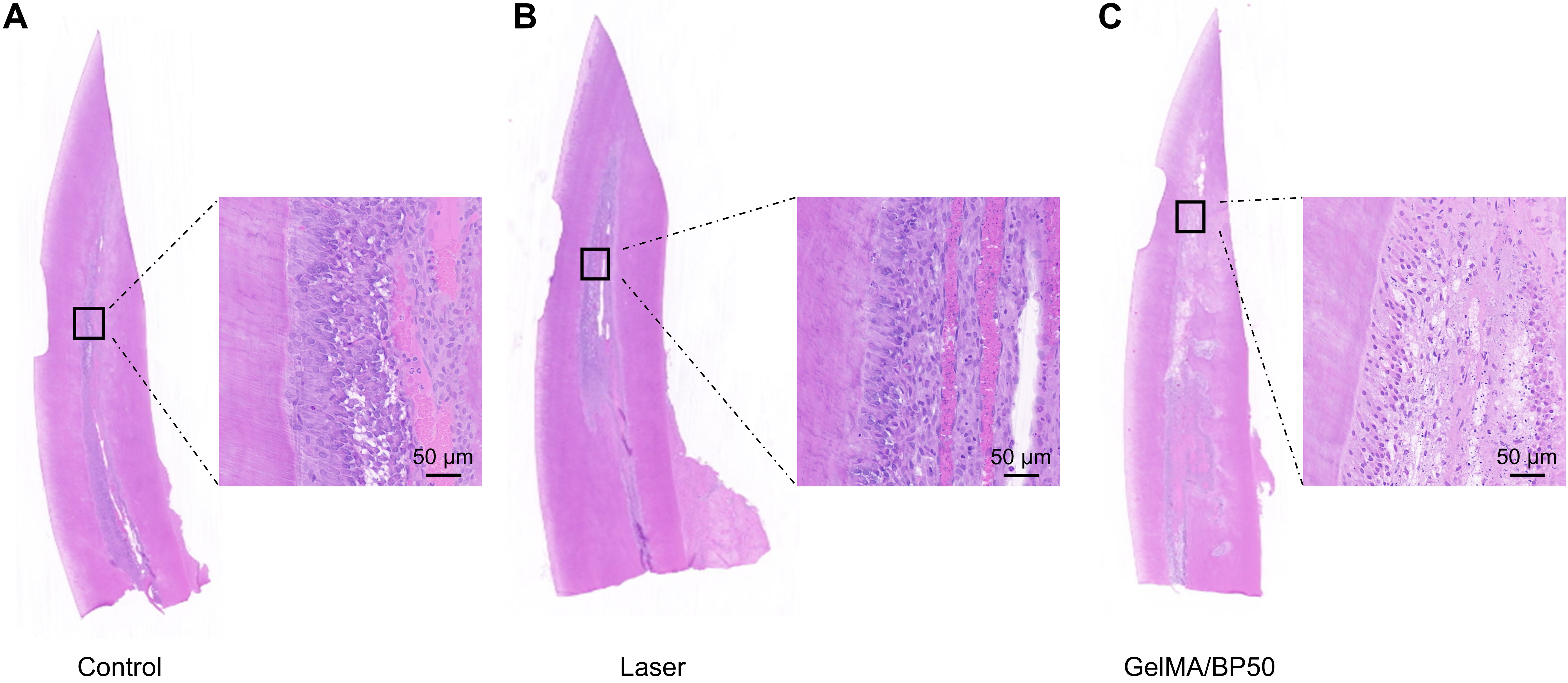
**

**Figure S5.** H&E images of rat's dental pulp tissue. A) Control group, B) Laser group, C) GelMA/BP50 group.
